# Rare Case of Growth Hormone Insensitivity Syndrome Correlated With Hypothyroidism: A Case Report

**DOI:** 10.7759/cureus.62575

**Published:** 2024-06-17

**Authors:** Sri Sita Naga Sai Priya K, Jayant D Vagha, Sham Lohiya, Keta Vagha, SreeHarsha Damam, Rahul Khandelwal, Chaitanya Kumar Javvaji, Siddhartha Murhekar

**Affiliations:** 1 Pediatrics, Datta Meghe Institute of Higher Education and Research, Wardha, IND; 2 Trauma and orthopaedics, East Kent Hospitals University NHS Foundation Trust, Canterbury, GBR

**Keywords:** short stature, hormone replacement therapy, igf-1, growth hormone, hypothyroidism

## Abstract

Growth hormone insensitivity syndrome (GHIS) is a rare genetic disorder characterized by short stature due to the body's inability to effectively utilize growth hormone (GH). This case report describes a patient with concurrent hypothyroidism and GHIS. This patient is an 11-year-old female presented with short stature; general examination suggested a prominent forehead and a depressed nasal bridge. Laboratory evaluations revealed elevated thyroid-stimulating hormone (TSH) levels alongside low levels of triiodothyronine (T3) and thyroxine (T4), indicating hypothyroidism. Additionally, elevated GH levels and significantly reduced insulin-like growth factor 1 (IGF-1) levels confirmed the diagnosis of GHIS. The patient was managed with thyroid hormone replacement therapy and recombinant GH. This dual therapeutic approach will lead to improvements in both thyroid function and growth parameters. This case underscores the importance of recognizing and addressing coexisting endocrine disorders in patients with GHIS to optimize their growth and developmental outcomes. Early diagnosis and a comprehensive treatment strategy are essential for managing such complex cases effectively.

## Introduction

Growth hormone insensitivity syndrome (GHIS), marked by a lack of responsiveness to growth hormone (GH), presents with stunted growth after birth and significantly reduced levels of insulin-like growth factor 1 (IGF-I) in the bloodstream, despite the body producing higher amounts of GH. It primarily follows an autosomal recessive inheritance pattern.

The clinical manifestations of GHIS closely resemble those seen in individuals with GH deficiency, accompanied by biochemical markers indicating GH resistance [1]. GHIS has an overall prevalence of 1-9 per 1,000,000 individuals [2]. In 1959, Zvi Laron identified the syndrome in three siblings from a Jewish family with a common ancestry, all displaying pronounced short stature. Despite resembling individuals with hypopituitarism, these siblings had remarkably elevated levels of serum human GH [3].

## Case presentation

An 11-year-old girl born out of non-consanguineous marriage presented with short stature. She had a height of 108 cm and a weight of 13 kg, indicating a height less than the third centile as per CDC charts, with a BMI of 11.1 kg/m^2^ lying below the third centile as per CDC charts. Upper segment:lower segment ratio was 0.9:1. Arm span suggestive of 102 cm. Mid-parental height was 155 cm lying between the 50th to 75th centile as per the CDC chart. Her height age was five years, and her bone age was nine years. The patient is at the prepubertal stage of Tanner's development. Examination revealed an active child with a flat nasal bridge and short neck. All her vitals and systemic examination were found to be normal. Tier one investigations for short stature included a hemogram with erythrocyte sedimentation rate, urine and stool analysis, kidney function test, liver function test, calcium, phosphate, and random blood sugar were suggestive of normal, hence proceeded with tier two investigations which included thyroid profile and karyotyping. Thyroid function tests showed low levels of triiodothyronine (T3), thyroxine (T4), and elevated levels of thyroid-stimulating hormone. The repeat thyroid test was conducted and yielded the same values as the previous one. Later on, the patient was started on tab Thyroxine with 4 μg/kg/day and then proceeded with tier three investigations, including follicle-stimulating hormone (FSH), luteinizing hormone (LH), calcium, tissue transglutaminase antibody-immunoglobulin G (tTG-IgG), and tissue transglutaminase antibody-immunoglobulin A (tTG-IgA). All these investigations are suggestive of normal. An MRI of the brain was performed because of short stature, and the findings are normal. Basal IGF-1 is 5 ng/ml, and GH level is 9 ng/ml. GH stimulation test done using clonidine revealed that GH levels at 30 min, 60 min, and 90 min were 22 ng/ml, 32.69 ng/ml, and 35.13 ng/ml which are higher than the normal range (0.03-4 ng/ml) and IGF-1 levels post 90 minutes of clonidine suggestive of 11.1 ng/ml (38-190 ng/ml). All the investigations of the child for short stature are shown in Table 1.

**Table 1:** Investigations for short stature

Laboratory investigations	Observed value	Reference range
Hemoglobin	12.2	13-15 g/dl
Total leucocyte count	8,000	4,000-11,000/cumm
Platelet	4,20,000	1,50,000-4,50,000/cumm
Urea	16	9-20 mg/dl
Creatinine	0.5	0.6-1.2 mg/dl
Sodium	135	137-145 mmol/l
Potassium	3.7	3.5-5.1 mmol/l
Alkaline phosphatase	112	38-126 unit/l
Alanine transaminase	36	<50 U/l
Aspartate transaminase	42	17-59 U/L
Total protein	7.2	6.3-8.2 g/dl
Albumin	4.1	3.5-5 g/dl
Total bilirubin	0.8	0.2-1.3 mg/dl
Unconjugated bilirubin	0.1	0-0.3 mg/dl
Conjugated bilirubin	0.2	0-1.1 mg/dl
Globulin	3.3	2.3-3.5 mg/dl
Tissue transglutaminase antibody-immunoglobulin G	14.1	<15I U/ml
Tissue transglutaminase antibody-immunoglobulin A	5.13	<8 IU/ml
Triiodothyronine	0.6	0.97-1.69 ng/ml
Thyroxine	3.6	5.53-11 μg/dl
Thyroid-stimulating hormone	11.7	0.46-4.68 μIU/ml
Follicle-stimulating hormone	2.09	<0.1-12 IU/I
Luteinizing hormone	4	<0.1-13.4 IU/I
Erythrocyte sedimentation rate	12	2-13 mm/h
Random blood sugar	98	<120 mg/dl
Calcium	8.8	8.4-10.4 mg/dl
Phosphate	3.4	2.5-4.5 mg/dl

The test for generating IGF-1 evaluates the body's capacity to produce IGF-1 when GH prompts it. A normal response to the test would be a significant increase in serum IGF-1 levels, indicating that the body is capable of producing IGF-1 in response to GH stimulation using clonidine at a dose of 150 μg/m^2^. However, in this case, following the administration of clonidine, the serum IGF-1 level persisted low 11.1 ng/ml, which is significantly below the normal range of 38-190 ng/ml. This indicates that the body is not able to generate sufficient amounts of IGF-1 even after GH stimulation, which is referred to as a failure of IGF-1 generation. Consequently, a diagnosis of partial GHIS was confirmed. The results of GH stimulation test are shown in Table 2. Treatment began with subcutaneous injections of GH at a dosage of 0.5 IU subcutaneously along with oral calcium and vitamin D supplements . The patient was deemed stable enough for discharge and was advised to continue treatment at home while attending regular follow-up appointments plan for follow-ups.

**Table 2:** Results of growth hormone stimulation test GH: growth hormone, IGF-1: insulin-like growth factor 1.

Parameter	Observed value	Reference range
Growth hormone (stimulation test)		
Basal GH	9 ng/ml	0.03-4.0 ng/ml
Basal IGF-1	5 ng/ml	38-190 ng/ml
Clonidine given		
30 min	22 ng/ml	0.03-4.0 ng/ml
60 min	32.69 ng/ml	0.03-4.0 ng/ml
90 min	35.13 ng/ml	0.03-4.0 ng/ml
IGF-1	11.1 ng/ml	38-190 ng/ml

## Discussion

The patient exemplifies a partial case of showing GHIS. Unfortunately, the diagnosis and treatment were initiated at a later age. Clinically, patients with GHIS resemble those with severe GH deficiency. The patient's birth history was normal, including normal birth weight. Over time, the patient developed short stature, which went unnoticed until the present age. It is an uncommon genetic condition that stems from a mutation in the GH receptor gene found on chromosome 5's short arm. This defect leads to an inability of the body to respond to GH, which in turn causes growth failure and other symptoms that resemble isolated GH deficiency type 1A [3].

Children affected with GHIS and those with isolated GH deficiency present with similar clinical features. The GH receptor, which is essential for GH function, is determined by a gene located on chromosome 5's short arm (5p13-p12). GHIS can result from different homozygous point mutations occurring in the GH receptor gene [4]. These mutations, particularly in the extracellular domain of the receptor, disrupt GH binding and lead to GH insensitivity [5]. The fundamental metabolic defect in primary GHIS is the failure of target organs to respond to endogenous GH. In 1984, liver biopsies revealed that GH fails to bind to its receptors, preventing the generation of IGF-1 [6].

Comparable to children experiencing GH deficiency, individuals with GHIS are susceptible to hypoglycemia not only during the newborn period but also throughout childhood and adulthood [7]. Puberty is delayed in children with GHIS, with boys experiencing a greater delay than girls, and neither gender undergoes the typical pubertal growth spurt. However, despite these delays, both sexes reach full sexual maturity and do not face reproductive challenges in early adulthood. The impact of GH on the skeletal system is well established; without GH action, skeletal maturation is delayed. As a result, the epiphyses of long bones close later; typically, girls experience this phase between the ages of 16 and 18, while boys go through it between 20 and 22 years old [8].

Other potential complications include delayed puberty, reduced muscle mass and strength, increased body fat, and decreased bone density, which can lead to osteopenia or osteoporosis. Fractures are another potential complication of GH insensitivity, as reduced bone density can increase the risk of bone fractures. However, all the above features were not present in this case which is pointing toward partial GHIS. [9]. In summary, the diagnostic criteria for GHIS involve the assessment of multiple parameters related to GH and IGF-1 activity in the body, including measurements of height, GH and IGF-1 levels, Insulin-like growth factor binding protein 3 (IGFBP-3) levels, and GH binding. In our case, the criteria for identifying GHIS included height below the third percentile, a basal GH level greater than 2.5 µg/L, a basal IGF level below 50 µg/L, and an IGF-1 generation test indicating levels below 15 µg/L, with prepubertal stage of development. According to these criteria, only four features point toward a diagnosis of GHIS. Diagnosis of GHIS is considered if a significant deficiency or impairment is detected in multiple parameters [10].

In this case, the patient displayed short stature and insufficient weight gain. Laboratory evaluation revealed a bone age of nine years, which was significantly advanced compared to a height age of five years. The presence of growth hormone resistance can explain the lack of growth, affecting both her height and weight. Concurrently, elevated levels of TSH indicated hypothyroidism. After thorough examinations across all three tiers, the diagnosis of partial GHIS was confirmed by an endocrinologist. This syndrome is marked by elevated levels of GH despite impaired growth, which was supported by diminished IGF levels following clonidine administration during the GH stimulation test. The patient commenced a trial of recombinant GH therapy and was scheduled for follow-up appointments. However, due to financial limitations, genetic studies and IGF injections were postponed at that point.

## Conclusions

Short stature can indeed be a manifestation of isolated GH deficiency, GH resistance syndrome. While evaluating a patient of short stature, the first step is to conduct a thorough medical history and physical examination, which can provide valuable information about potential underlying causes of the patient’s growth delay. If the level one and two investigations do not reveal any significant underlying abnormality, then level three investigations may be considered. Level three investigations are more specialized and may include more complex molecular tests to look for rare or specific causes of short stature.
